# CMAS Corrosion Behavior of Mid-Entropy Rare-Earth Hafnate (Y_0.3_Gd_0.3_Yb_0.4_)_4_Hf_3_O_12_ as Thermal Barrier Coating Candidate

**DOI:** 10.3390/ma17235892

**Published:** 2024-12-01

**Authors:** Fuxing Ye, Yuan Yao, Fanwei Meng, Tianyuan Luo

**Affiliations:** 1 Tianjin Key Laboratory of Advanced Joining Technology, School of Materials Science and Engineering, Tianjin University, Tianjin 300072, China; yuanyao0914@163.com (Y.Y.); fwmeng@tju.edu.cn (F.M.); luotianyuan@huawei.com (T.L.); 2 Key Laboratory of Advanced Ceramics and Machining Technology of Ministry of Education, Tianjin 300072, China

**Keywords:** mid-entropy ceramic, thermal barrier coatings, CMAS corrosion mechanism, ultrafast high-temperature sintering

## Abstract

High-temperature CMAS corrosion has become a crucial factor inhibiting the further development of thermal barrier coatings (TBCs) because of the increasing service temperature of aero-engines. Herein, a novel mid-entropy rare-earth hafnate (Y_0.3_Gd_0.3_Yb_0.4_)_4_Hf_3_O_12_ (YGYbH) was prepared by ultrafast high-temperature sintering (UHS) technology, and its CMAS corrosion behavior and mechanism were investigated. During corrosion, the Ca_2_RE_8_(SiO_4_)_6_O_2_ apatite phase with a lower formation enthalpy and entropy-stabilized effect had a more intense tendency to be generated, which improves the density and stability of the reaction layer, hindering the further penetration of molten CMAS. Moreover, the significant lattice distortion caused by the rare-earth ions with different radii impeded the ionic diffusion, which delayed the CMAS corrosion reaction. In general, YGYbH, with excellent CMAS corrosion resistance, has the potential to serve as a next-generation TBC material.

## 1. Introduction

In order to improve the thrust-to-weight and operational efficiency of aero-engines, ultrahigh service temperature has become a crucial factor in destroying the stable service of the superalloy substrate [1,2]. Thermal barrier coatings (TBCs) are engineered to shield superalloy substrates in aero-engines from extreme thermal environments by leveraging materials with outstanding mechanical properties, thermophysical properties, high-temperature stability, and corrosion resistance [3,4]. Additionally, aero-engines will inevitably ingest and deposit particles such as sand, runway debris, and volcanic ash on the blade surface during service [5,6]. Under high temperatures, the deposit is melted to form a melt (CMAS) composed mainly of CaO, MgO, AlO_1.5_, and SiO_2_, which infiltrates and corrodes the matrix through pores and cracks by the capillary action [7]. Traditional 6–8 wt.% Y_2_O_3_ stabilized ZrO_2_ (YSZ) is typically used to prepare TBC due to its excellent performance [8,9]. However, the metastable tetragonal (t′) YSZ will transform into tetragonal (t) and cubic (c) phases at higher temperatures (≥1200 °C), and the cubic (c) YSZ will transform into monoclinic (m) phase during cooling. Severe phase transition with significant volume change and poor sintering resistance have become two main factors causing the premature failure cracking of YSZ coating [10,11]. Except for the above factors, YSZ is easily chemically corroded by CMAS melt to form silicate, anorthite, and others, resulting in premature failure [12,13]. Therefore, developing next-generation TBC materials with excellent resistance to CMAS corrosion to replace YSZ has become an urgent research topic.

In recent years, the concept of entropy has been introduced into the design and development of ceramic material, and TBC ceramics with entropy-stabilized effects are proven to exhibit more excellent properties than single-component TBC ceramics [14,15,16,17]. Rost et al. [18] initially succeeded in preparing high-entropy oxide ceramics (Mg_0.2_Ni_0.2_Co_0.2_Cu_0.2_Zn_0.2_)O and demonstrated that configurational disorder caused by populating a single sublattice with distinct cations induced the entropy-stabilized effect. Tu et al. compared the CMAS corrosion resistance of high-entropy rare-earth zirconate (La_0.2_Nd_0.2_Sm_0.2_Eu_0.2_Gd_0.2_)_2_Zr_2_O_7_ with single-component La_2_Zr_2_O_7_, and the result indicates that the (La_0.2_Nd_0.2_Sm_0.2_Eu_0.2_Gd_0.2_)_2_Zr_2_O_7_ exhibited better CMAS corrosion resistance and the corrosion products with a fine microstructure which prevent the formation of cracks [16]. Guo et al. designed a high-entropy rare-earth disilicate (Lu_0.2_Yb_0.2_Er_0.2_Tm_0.2_Sc_0.2_)_2_Si_2_O_7_, and the study showed that the high-entropy disilicate ceramic performs good phase stability, lower coefficient of thermal expansion and thermal conductivity, and better water vapor corrosion resistance, attributed to the multiple doping effects [17]. In addition, some scholars have found that rare-earth hafnates with entropy-stabilized effects display excellent resistance to CMAS corrosion at high temperatures [7,18]. Ye et al. studied the CMAS corrosion behavior of the high-entropy rare-earth hafnate (La_0.2_Nd_0.2_Sm_0.2_Eu_0.2_Gd_0.2_)_2_Hf_2_O_7_ and demonstrated that the dense corrosion products layer acts as a barrier to the penetration of molten CMAS [19]. Furthermore, in our previous research [20], a mid-entropy rare-earth hafnate ceramic with a composition of (Y_0.3_Gd_0.3_Yb_0.4_)_4_Hf_3_O_12_ (YGYbH) was designed and successfully prepared by the UHS technology, and it possessed excellent high-temperature phase stability, low thermal conductivity, and high thermal expansion coefficient matched to superalloy substrate, which exhibits developability. However, the high-temperature CMAS corrosion behavior of YGYbH hafnate has not been assessed.

In this work, the high-temperature CMAS melt corrosion behavior of YGYbH at 1300 °C and 1400 °C was studied, and the CMAS corrosion mechanism was analyzed. The YGYbH hafnate demonstrates exceptional resistance to high-temperature CMAS corrosion, indicating its strong potential as a next-generation material for thermal barrier coatings.

## 2. Materials and Methods

### 2.1. The Preparation of Samples

**Figure 1:**
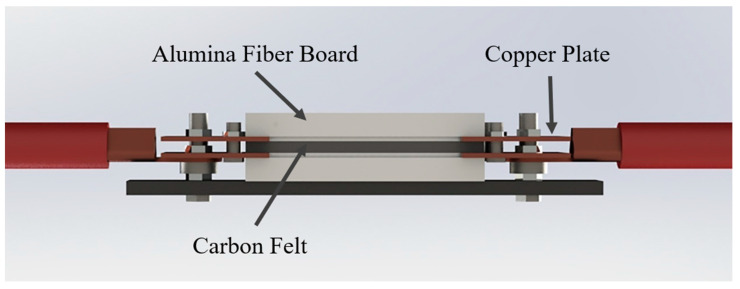
Schematic illustration of the UHS equipment [7].

To fabricate the ceramic bulks with the composition of (Y_0.3_Gd_0.3_Yb_0.4_)_4_Hf_3_O_12_, oxide powders (Y_2_O_3_, Gd_2_O_3_, Yb_2_O_3,_ and HfO_2_, 99.9%, Beijing HWRK Chen Co., Ltd., Beijing, China) were preheated at 800 °C for 2 h to eliminate crystal water. The powders were then weighted according to the molar ratio, followed by ball-milling at 400 rpm for 15 h. The subsequent slurry was dried and passed through a 200-mesh sieve to obtain the fine YGYbH powder. And 0.5 g of the mixed powder was weighed and pressed by cold isostatic pressing to generate the green ceramic bulks (Φ15 mm × 0.5 mm) [20]. The mid-entropy ceramic YGYbH was subsequently prepared using UHS technology, with a schematic illustration of the UHS equipment shown in Figure 1 [7]. Detailed sintering densification and operation procedures can be found in our previous study [20].

### 2.2. CMAS Corrosion Behavior Experiment

For high-temperature corrosion experiments, the CMAS powder with a mole ratio of 33CaO-9MgO-13AlO_1.5_-45SiO_2_ (at. %) was prepared to simulate the average composition of deposits on the blade surface [21]. To eliminate the crystal water, the original CaO, MgO, Al_2_O_3,_ and SiO_2_ powders (99.9%, Tianjin Jiangtian Chemical Technology Co., Ltd., Tianjin, China) were preheated at 800 °C for 4 h. The dried powders were then weighted in the desired molar ratio and ball-milled at 400 rpm for 4 h. After being dried at 100 °C for 6 h, the mixture was heated at 1500 °C for 1 h to produce a CMAS melt, which was subsequently quenched by deionized water to form the CMAS glass. Finally, the CMAS glass was ground and sieved through a 200-mesh sieve to obtain the fine CMAS powder. For the high-temperature corrosion experiment, the surface of the YGYbH ceramic bulk is uniformly covered by the fine CMAS powder at 20 mg/cm^2^ loading. Ceramic samples covered with CMAS powder are heated in a muffle furnace at 1300 °C and 1400 °C for 0.5 h, 1 h, 2 h, 5 h, and 10 h to conduct the CMAS corrosion experiments. Moreover, the CMAS powder was mixed with YGYbH powder (obtained after grinding) at a mass ratio of 0.3:1, followed by cold isostatic pressing to obtain the bulks. The bulks were exposed to the same high-temperature conditions as the corrosion experiments for subsequent detection of corrosion products.

### 2.3. Characterization Methods

In this work, Archimedes’ Method was used to measure the density of YGYbH ceramic bulks. X-ray diffraction (XRD, D8 Advance, Bruker AXS, Karlsruhe, Germany) was applied to detect the phase structure of the corrosion products. The cross-sectional morphology and elemental distribution were characterized by a scanning electron microscope (SEM, Hitachi S4800, Tokyo, Japan) equipped with energy-dispersive spectroscopy (EDS, X-MAX20, Oxford, UK).

### 2.4. Theoretical Calculation Methods

The first-principles calculations were performed to investigate the corrosion products formed when YGYbH is corroded by CMAS melt. On the basis of Density Functional Theory (DFT), all calculations were executed by the Vienna Ab-initio Simulation Package (VASP-6.1.2) [22]. The Projector Augmented Wave (PAW) methodology was applied to model the intricate interactions of electron–ion, and Generalized Gradient Approximation (GGA) was employed to delineate the electron exchange–correlation potentials [23,24]. In the calculations, the plane wave cutoff energy was set to a default value of 500 eV. The convergence criterion of structural optimization and the self-consistent field was 1 × 10^−6^, and the maximum stress of each atom was 0.01 eV/atom. The formula for the formation of enthalpy is as follows:

(1) $$H_{formation}=\frac{E_{A_{l}B_{m}}-lE_{crystal}^{A}-mE_{crystal}^{B}}{l+m}\;\;$$

where $H_{formation}$ is the formation enthalpy, and l and m present the number of *A* and *B* atoms in *A_l_B_m_* crystal, respectively. $E_{crystal}^{A}$ and $E_{crystal}^{B}$ present the energy of a single atom in *A_l_B_m_* crystals.

## 3. Results and Discussion

### 3.1. Characterization of Mid-Entropy YGYbH Hafnate Prepared by UHS

**Figure 2:**
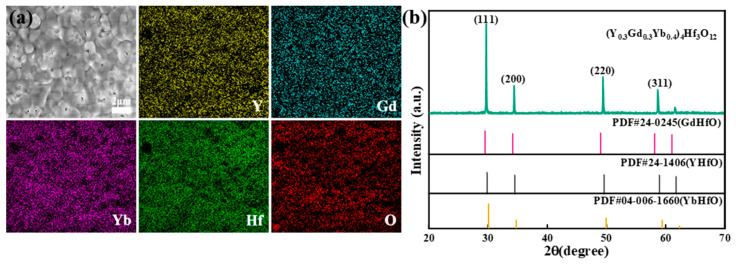
(**a**) SEM-EDS mapping of YGYbH; (**b**) XRD pattern of sintered YGYbH ceramic bulks, and standard PDF cards of Gd_2_Hf_2_O_7_, Y_2_Hf_2_O_7_ and Yb_2_Hf_2_O_7_.

The microstructure, element distributions, and XRD pattern of the sintered YGYbH ceramic bulks are presented in Figure 2. The elements of RE, Hf, and O are uniformly distributed, and no precipitation of second-phase impurities was found, demonstrating that YGYbH bulk has a uniform chemical composition, as shown in Figure 2a. The average grain size of the YGYbH bulk is measured by the linear intercept method, and the result shows that the YGYbH bulk exhibits a fine and dense microstructure, with an average grain size of approximately 1.19 µm. The porosity (*φ*) of the sintered ceramic bulks was calculated using the Equation (2):

(2) $$\varphi =1-\rho /\rho_{0}$$

where ρ represents the bulk density (measured as 8.35 g/cm^3^ using Archimedes’ method), and $\rho_{0}$ is the theoretical density (calculated as 8.69 g/cm^3^). Based on these values, the porosity was found to be 3.91%, indicating that the YGYbH fabricated using UHS technology achieves remarkable densification. Moreover, the phase structure analysis (Figure 2b) reveals diffraction peaks corresponding exclusively to the fluorite structural planes (111), (200), (220), and (311). This confirms that a single-phase fluorite structure was successfully synthesized in the YGYbH ceramic.

### 3.2. CMAS Corrosion Behavior of YGYbH at High Temperatures

#### 3.2.1. CMAS Corrosion Behavior of YGYbH at 1300 °C

**Figure 3:**
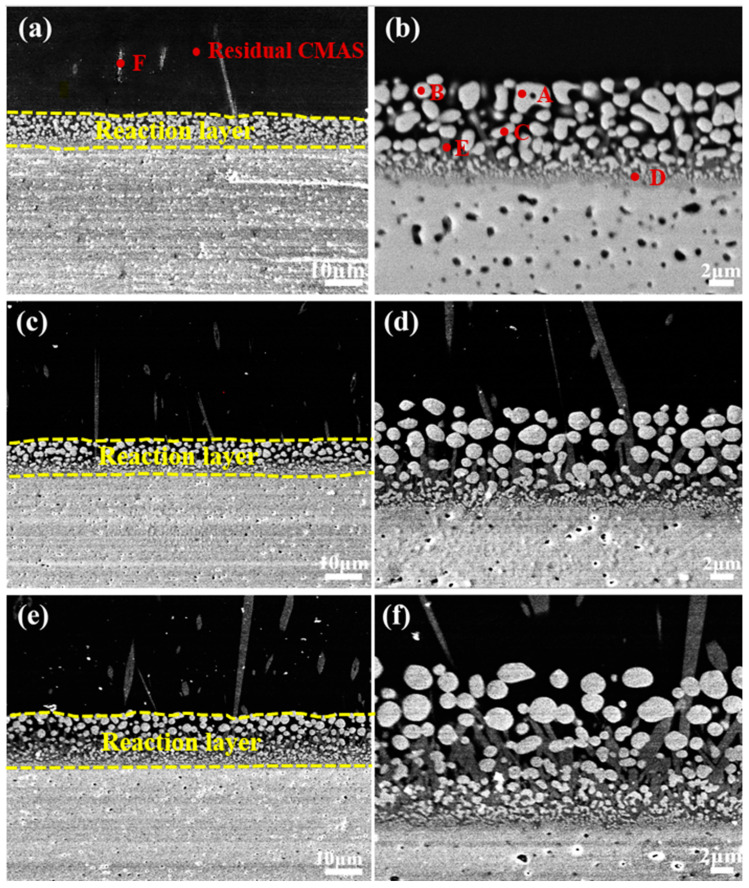
Cross-sectional morphologies of YGYbH after CMAS melt corrosion at 1300 °C for 0.5 h (**a**,**b**), 1 h (**c**,**d**), and 2 h (**e**,**f**).

**Figure 4:**
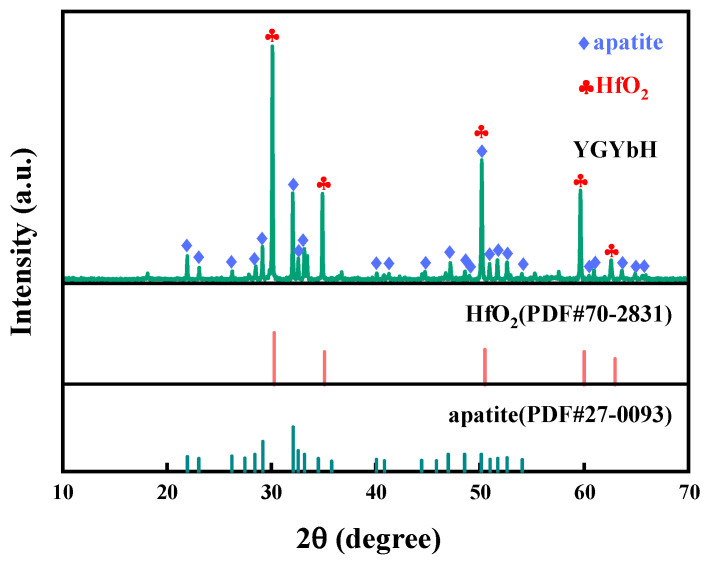
XRD pattern of CMAS corrosion products at 1300 °C for 10 h, and standard PDF cards of HfO_2_ and apatite.

The CMAS corrosion behavior of the YGYbH at 1300 °C for 0.5 h, 1 h, 2 h, 5 h, and 10 h are investigated, and the cross-section morphology is shown in Figure 3. After being exposed for 0.5 h, the YGYbH was corroded by the CMAS melt, and a reaction layer with a thickness of only 8 µm can be observed. From the high-magnification view of the reaction layer (Figure 3b), it can be seen that it is mainly made up of a light phase and a gray phase. The light contrast phase contains three forms: a spherical morphology with holes (A), a spherical morphology (B), and a complete rodlike morphology (C); meanwhile, the gray contrast phases consist of a small amount of bulk-shaped morphology (D) and rodlike morphology (E). Above the reaction layer, a few floating tiny acicular gray phases (F) can be observed, and the chemical compositions of different morphologies are presented in Table 1. The light phases predominantly consist of Hf and O elements in a stoichiometric ratio of about 1:2, and the gray morphologies mainly consist of Ca, Si, rare-earth elements (Y, Gd, Yb), and O elements. Combining the XRD patterns of corrosion products at 1300 °C (Figure 4), it can be identified that the light phase is the HfO_2_ phase with fluorite structural phase, and the gray phase is the apatite phase Ca_2_RE_8_(SiO_4_)_6_O_2_ (RE = Y, Gd, and Yb). As a single-phase solid solution, the apatite phase Ca_2_RE_8_(SiO_4_)_6_O_2_ contains three rare-earth elements (Y, Gd, and Yb) in similar proportions as YGYbH, indicating that the apatite phase also possesses the entropy-stabilized effect.

With the corrosion time prolonged to 1 h, the reaction layer grew to a thickness of 11 µm, as shown in Figure 3c,d. Compared with the morphologies at 0.5 h, the HfO_2_ phase was predominantly observed in the spherical morphology (B), while the spherical morphology with holes (A) disappeared. Furthermore, there was also an increase in both the number and size of gray apatite phases (D, E) and floating acicular apatite (F). For the case of 2 h, the reaction layer thickness increases to 16 µm (Figure 3e,f), with the HfO_2_ phase becoming more spherical and the number of apatite phases increasing. Additionally, a denser reaction layer began to form as the continuity of the apatite phases improved at the bottom of the layer. This enhanced continuity contributed to greater structural stability, effectively hindering further CMAS penetration.

**Figure 5:**
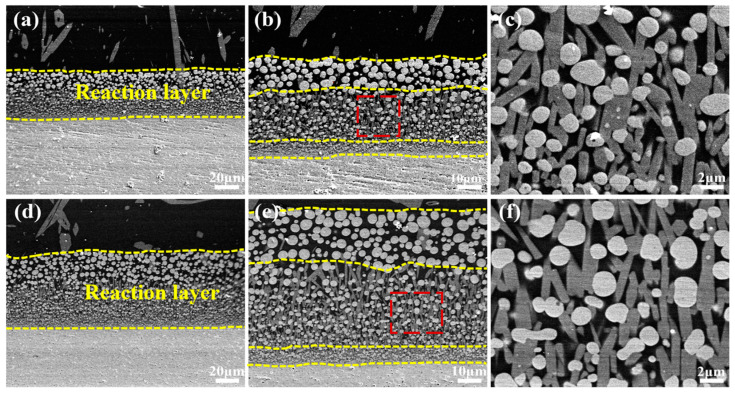
Cross-sectional morphologies of YGYbH after CMAS melt corrosion at 1300 °C for 5 h (**a**–**c**) and 10 h (**d**–**f**).

When the reaction time is extended to 5 h, the reaction layer’s thickness significantly increases to 42 µm, and its structure undergoes noticeable changes compared with 2 h. As shown in Figure 5a–c, it can be seen from the high magnification of the reaction layer that the whole reaction layer is clearly made up of three layers from top to bottom: (1) the upper layer consisting of the remotely distributed spherical HfO_2_ phase; (2) the intermediate layer containing the evenly arranged rodlike apatite phase and approximately spherical HfO_2_ phase; and (3) the bottom layer exhibiting a dense structure. It can be seen from Figure 5b that the density of the reaction layer increases from the upper layer to the bottom layer. As the corrosion time increased to 10 h, the thickness of the reaction layer increased to 72 µm, obviously thinner than the most widely used TBC material YSZ, of which the reaction layer thickness reaches 70 µm after only 15 min of corrosion at 1250 °C [25]. As seen in Figure 5d–f, the increased thickness of both the upper and intermediate layers contributes to the overall thickening of the reaction layer. And from Figure 5b,e, with the corrosion time prolonged, the density of the bottom layer with protective properties increases. The size of the HfO_2_ phase in the upper layer grows significantly twice that of 5 h, while the HfO_2_ and apatite phases in the intermediate layer grow indistinctively compared with the case of 5 h. And Figure 5c,f is the high magnification of the intermediate layer, which shows that a number of rodlike apatites fill the gaps between the HfO_2_ phases, further enhancing the reaction layer’s density. Generally, a dense structure consisting of homogeneously distributed HfO_2_ and apatite phases is formed at the bottom of the reaction layer for both 5 and 10 h at 1300 °C, which is conducive to preventing further penetration of CMAS melt.

**Figure 6:**
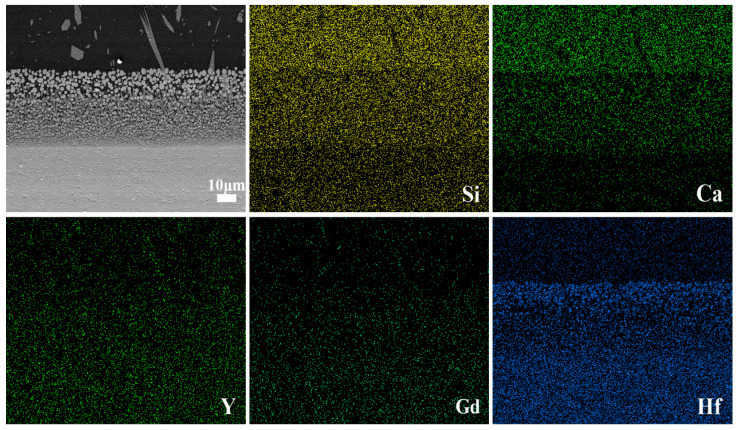
Cross-sectional morphologies of YGYbH and the EDS mapping of Si, Ca, Y, Gd, and Hf elements after CMAS melt corrosion at 1300 °C for 10 h.

Figure 6 presents the cross-sectional element distribution of Si, Ca, Y, Gd, and Hf elements after CMAS melt corrosion at 1300 °C for 10 h. It can be observed that the elements Si and Ca, representing CMAS, are primarily distributed above the reaction layer. In contrast, the elements Y, Gd, and Hf, representing YGYbH, mainly exist below the reaction layer. The different element distribution between above and below the reaction layer indicates that the YGYbH possesses the ability to maintain its structural integrity under the attack of molten CMAS, illustrating the reaction inertness between YGYbH and CMAS melt. Therefore, the YGYbH hafnate is a promising next-generation TBC material.

#### 3.2.2. CMAS Corrosion Behavior of YGYbH at 1400 °C

**Figure 7:**
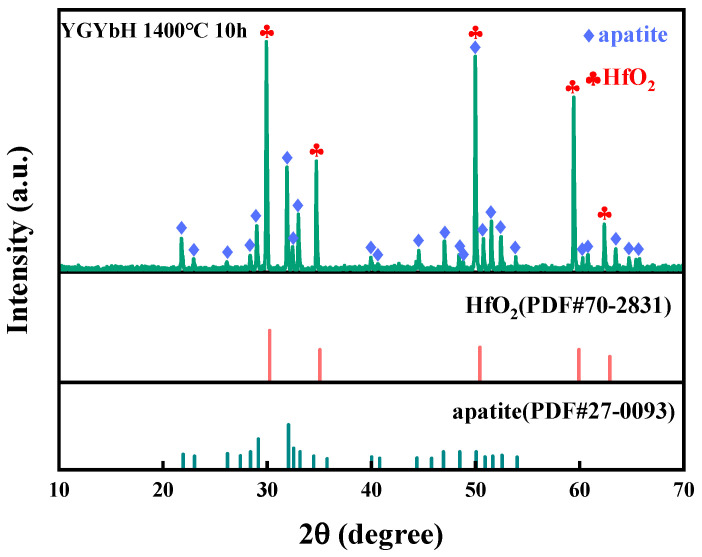
XRD pattern of CMAS corrosion products at 1400 °C for 10 h, and standard PDF cards of HfO_2_ and apatite.

The corrosion products of YGYbH exposed to CMAS melt at 1400 °C for 10 h were analyzed, as shown in Figure 7. The corrosion products observed at 1400 °C were similar to those formed at 1300 °C, consisting of the HfO_2_ phase and the apatite phase Ca_2_RE_8_(SiO_4_)_6_O_2_. This indicates that the corrosion reaction mechanism remains unchanged despite the increase in temperature. Therefore, the CMAS corrosion behavior of YGYbH at 1400 °C can be interpreted using the results obtained at 1300 °C.

**Figure 8:**
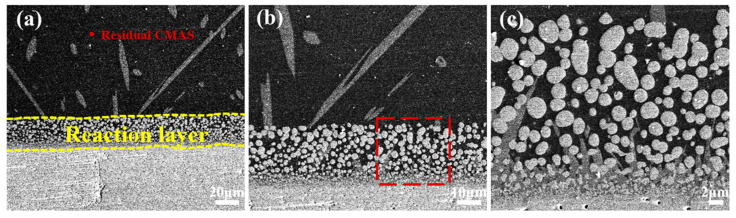
Cross-sectional morphologies of YGYbH after CMAS melt corrosion at 1400 °C for 0.5 h (**a**–**c**).

The CMAS corrosion behavior of the YGYbH at 1400 °C for 0.5 h, 1 h, 2 h, and 5 h are studied. As shown in Figure 8a–c, the reaction layer develops with a thickness of 25 µm after being reacted with CMAS melt for 0.5 h, which is markedly thicker than that observed at 1300 °C (8 µm). High-magnification cross-sectional images of the reaction layer reveal that it primarily consists of a light spherical HfO_2_ phase and a gray apatite phase, similar to the corrosion products formed at 1300 °C. Furthermore, the size and number of the floating apatite phase obviously increase compared with the same corrosion time case at 1300 °C, proving that the corrosion reaction rate significantly increases with the increase in reaction temperature.

**Figure 9:**
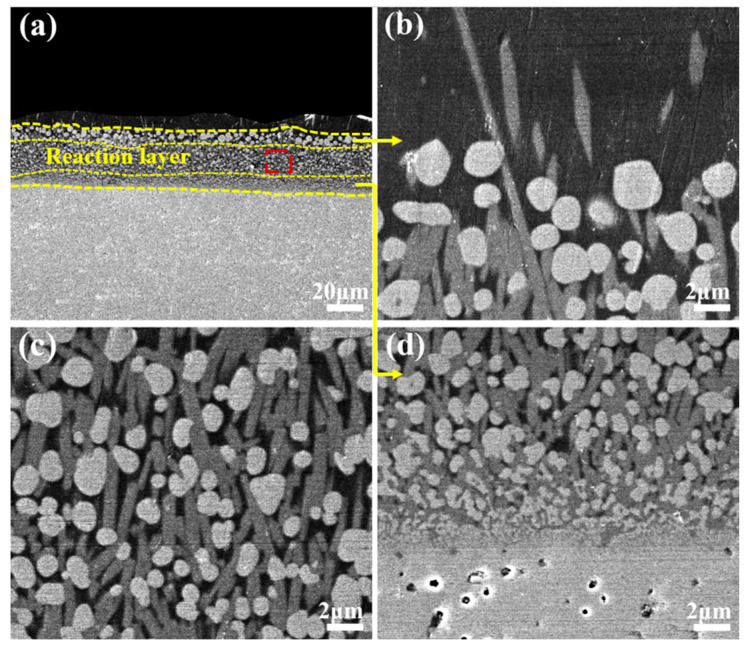
Cross-sectional morphologies of YGYbH after CMAS melt corrosion at 1400 °C for 1 h (**a**–**d**).

After 1 h of corrosion (Figure 9), a slight change in the thickness of the reaction layer is observed (27 µm). However, the structure of the reaction layer changes, and it can be categorized into three distinct layers like the case of 5 h at 1300 °C: (1) the upper layer comprising of the remotely arranged spherical HfO_2_ phase; (2) the intermediate layer containing the evenly distributed rodlike apatite phase and approximately spherical HfO_2_ phase; and (3) the bottom layer exhibiting the dense structure. From Figure 9d, it can be observed that the growth of the apatite phase and HfO_2_ phase contributes to the development of the dense bottom layer, indicating the capability of YGYbH to impede the penetration of CMAS melt.

**Figure 10:**
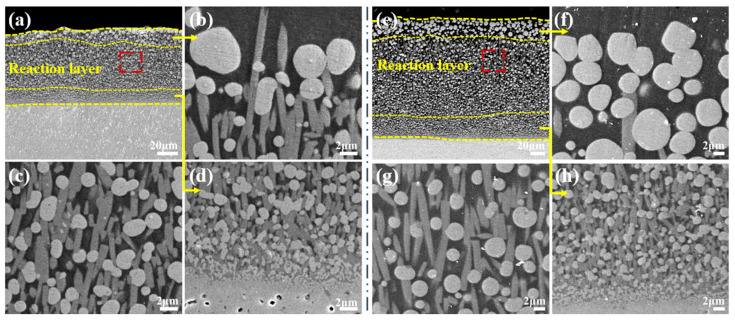
Cross-sectional morphologies of YGYbH after CMAS melt corrosion at 1400 °C for 2 h (**a**–**d**) and 5 h (**e**–**h**).

Upon further prolonging the corrosion time to 2 h, as shown in Figure 10a, the reaction layer is characterized by the three-layer structure, the thickness of which grows significantly to 70 µm, mainly due to the thickness growth of the intermediate layer. It can be seen from the magnification view in Figure 10b–d that the HfO_2_ phase becomes more rounded, and the bottom layer grows denser compared with that of 1 h. After being corroded by the CMAS melt for 5 h, the overall thickness of the reaction layer increases to 170 µm, maintaining the same three-layer structure observed for 1 h case at 1400 °C. As presented in Figure 10e–h, there is no significant change in the thickness of the upper and bottom layers; however, similar to the 2 h observation, the intermediate layer makes an essential contribution to the overall thickness increase.

### 3.3. Mechanism of the Chemical Reactions Between CMAS Melt and YGYbH

According to the description and analysis of the corrosion behavior of YGYbH under different reaction conditions, it can be concluded that the corrosion mechanism between CMAS melt and YGYbH is chemical reaction corrosion [19,26]. During corrosion, the two corrosion products of the HfO_2_ phase with a fluorite structural phase and the apatite phase Ca_2_RE_8_(SiO_4_)_6_O_2_ (RE = Y, Gd and Yb) are generated, which are insensitive to the increase in temperature. The equation for corrosion reaction is as follows:

(3) $$2YGYbH+2CaO\left(CMAS\right)+6SiO_{2}\to {Ca}_{2}{RE}_{8}{\left(SiO_{4}\right)}_{6}O_{2}+{6HfO}_{2}$$

**Figure 11:**
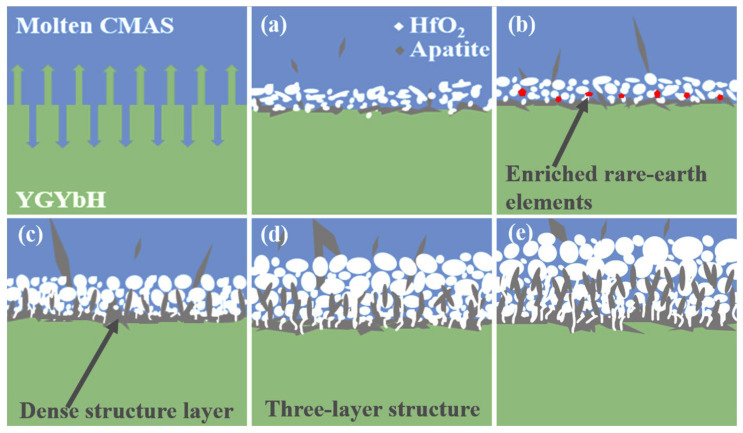
Schematic illustration of high-temperature CMAS corrosion mechanism with time prolonging from (**a**–**e**).

Figure 11 shows the schematic illustration of the corrosion behavior between YGYbH and CMAS melt at high temperatures. With the temperature increasing, the CMAS powder is molten and spread across the surface of the YGYbH ceramic matrix, and the corrosion reaction between the YGYbH and the CMAS melt proceeds through dissolution/re-precipitation. As the corrosion reaction processes, the corrosion product HfO_2_ phase gradually grows spherical, and the volume of corrosion products increases. When the HfO_2_ phase layer forms a certain continuity, the growth of the apatite phase towards the molten CMAS will be hindered by the continuous HfO_2_ phase, which causes their stacked growth at the bottom of the reaction layer. Therefore, a dense and continuous structure layer is generated to resist the penetration of CMAS melt during the stack growth. As the reaction time is further increased, a three-layer structure in Figure 11d is formed: (1) the upper layer comprising of the sparsely arranged spherical HfO_2_ phase; (2) the intermediate layer containing the uniformly distributed rodlike apatite phase and spherical HfO_2_ phase; and (3) the bottom layer with the dense structure. Furthermore, the bottom layer becomes denser with the corrosion time prolonged.

**Figure 12:**
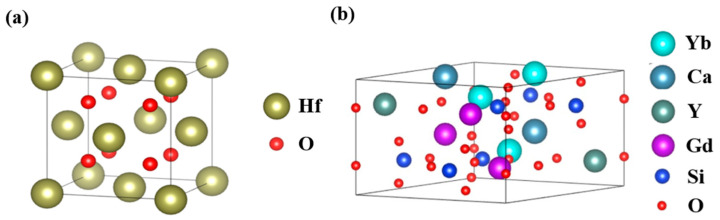
Simulated models of (**a**) HfO_2_ and (**b**) Ca_2_RE_8_(SiO_4_)_6_O_2_.

To further analyze the CMAS corrosion reaction mechanism of YGYbH, the first-principles calculations were conducted to determine the formation enthalpies of the two corrosion products and compare the formation tendency. Depending on the lattice structure, the Gamma k-points for HfO_2_ and Ca_2_RE_8_(SiO_4_)_6_O_2_ were 6 × 6 × 6 and 4 × 4 × 4, respectively, and the models of HfO_2_ and Ca_2_RE_8_(SiO_4_)_6_O_2_ were constructed by Materials Studio, as shown in Figure 12a,b. The more negative the formation enthalpy, the more favorable the formation of products. The resulting formation enthalpy of Ca_2_RE_8_(SiO_4_)_6_O_2_ is −343.900 kJ/mol, which is more negative than HfO_2_ (−121.002 kJ/mol). It can be concluded that the apatite phase Ca_2_RE_8_(SiO_4_)_6_O_2_ preferentially precipitates during the corrosion reaction. The rare-earth elements initially undergo a reaction with Ca^2+^ and Si^2+^ to generate the apatite phase Ca_2_RE_8_(SiO_4_)_6_O_2_, and the precipitation of the apatite phase inevitably accelerate the aggregation of Hf^4+^. Subsequently, when coming up to sufficient concentration, the Hf^4+^ is consumed to form the HfO_2_ phase. From the previous cross-sectional microstructures, it can be seen that the number of HfO_2_ phases is noticeably greater than that of the apatite phase due to the fact that 8 mol rare-earth ions in YGYbH are consumed to generate 1 mol apatite phase Ca_2_RE_8_(SiO_4_)_6_O_2_ [7]. In comparison, only 1 mol Hf^4+^ is consumed to produce 1 mol HfO_2_ phase [7].

**Figure 13:**
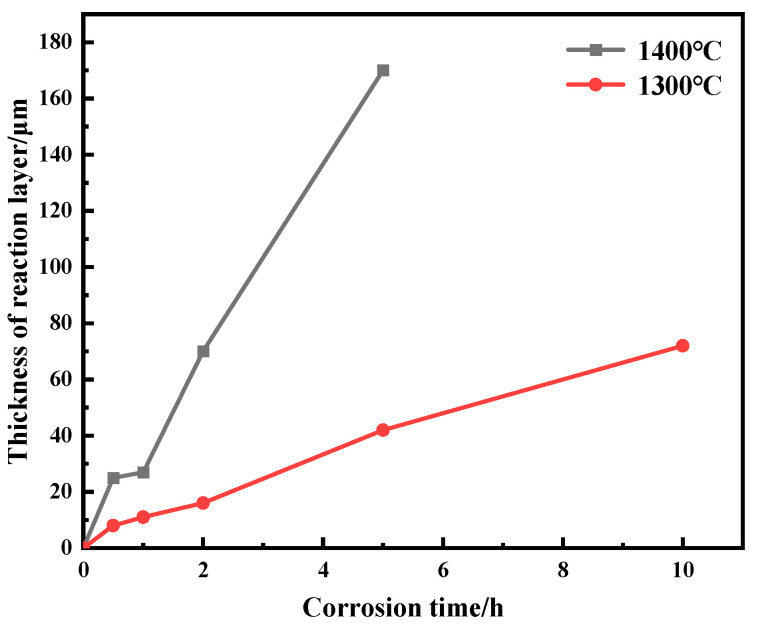
The variation curve of reaction layer thickness with corroded time at 1300 °C and 1400 °C.

As shown in Figure 13, the reaction layer thickness at 1300 °C is overall thinner than that at 1400 °C. According to Equation (4), the viscosity of the melt decreases exponentially with the increase in temperature.

(4) $$\eta =A\times \exp\left(B/T\right)\;\;\;\;$$

where *η* is the viscosity of the melt, *T* is the corrosion temperature, and *A* and *B* are the experimental measurement parameters. The exponentially decreasing viscosity of CMAS melt causes a more severe infiltration trend, and the higher temperature accelerates the corrosion reaction. Therefore, the CMAS corrosion at 1400 °C is more serious. Moreover, Wu et al. investigated the corrosion behavior of single-component La_2_Hf_2_O_7_ ceramic at 1300 °C, in which the CMAS penetration depth reached 209 µm at 1300 °C for 10 h [27], but the thickness of the corrosion reaction layer of the mid-entropy YGYbH ceramic is only 72 µm under the same corrosion conditions. For the entropy-stabilized material, the severe lattice distortion caused by the ionic radius difference inhibits the ionic diffusion, which restricts corrosion reactions, resulting in a lower corrosion reaction degree than a single-component material. In general, the performance of YGYbH against CMAS corrosion demonstrates that YGYbH has good resistance to CMAS corrosion at high temperatures, suggesting the excellent application prospect as a next-generation TBC material.

## 4. Conclusions

In this study, the CMAS corrosion behaviors of mid-entropy hafnate YGYbH at 1300 °C and 1400 °C were systematically investigated, and the CMAS corrosion mechanism of YGYbH was analyzed. The specific conclusions are summarized as follows:

(1) At high temperatures, the CMAS melt dissolves the YGYbH ceramic matrix, resulting in the formation of the HfO_2_ phase with a fluorite structural phase and the apatite phase Ca_2_RE_8_(SiO_4_)_6_O_2_ (RE = Y, Gd and Yb). During the corrosion, two corrosion products of apatite phase Ca_2_RE_8_(SiO_4_)_6_O_2_ with the entropy-stabilized effect and HfO_2_ phase are insensitive to the change in temperature. Moreover, the apatite phase with the entropy-stabilized effect can improve the stability of the corrosion reaction layer, which kinetically hinders the further penetration of the CMAS melt.
(2) Compared with HfO_2_ (−121.002 kJ/mol), the apatite phase has a lower formation enthalpy (−343.900 kJ/mol), suggesting that the apatite phase with lower formation enthalpy is preferentially generated under the dissolution/re-precipitation mechanism, which accelerates the aggregation of Hf^4+^ followed by the precipitation of HfO_2_ phase.
(3) With the increase in corrosion temperature and time, a dense structure layer is formed at the bottom of the reaction layer, which significantly restricts the growth of the reaction layer. Besides, the significant lattice distortion caused by the radius difference between the Y^3+^, Gd^3+,^ and Yb^3+^ of YGYbH reduces the ionic diffusion rate, further slowing the growth of the reaction layer. However, the decrease in the viscosity of the CMAS melt leads to an acceleration of the corrosion reaction as the temperature increases.

## Figures and Tables

**Table 1:** Elemental compositions of the CMAS corrosion products A-F of the YGYbH (in at. %).

at. %	Ca	Mg	Al	Si	Y	Gd	Yb	Hf	O
A	1.44	-	-	-	3.71	2.12	5.98	34.79	51.96
B	2.28	-	0.06	3.67	3.07	2.10	6.37	31.71	50.73
C	4.01	0.88	0.85	8.90	2.99	2.24	4.55	19.76	55.82
D	6.79	0.06	-	17.86	6.02	6.80	1.34	7.84	47.43
E	10.46	1.35	1.29	20.45	4.83	5.51	5.29	1.94	48.86
F	10.70	1.63	1.75	20.97	4.52	4.75	4.48	1.76	49.43

## Data Availability

The original contributions presented in this study are included in the article; further inquiries can be directed to the corresponding author.
